# Essential oil of *Piper hispidum* (Piperaceae) has efficacy against monogeneans, and effects on hematology and gill histology of *Colossoma macropomum*

**DOI:** 10.1590/S1984-29612024001

**Published:** 2023-12-11

**Authors:** Carliane Maria Guimarães Alves, Raimundo Rosemiro de Jesus Baia, Vitor Araújo Farias, Matheus Araújo Farias, Fernanda Layza Souza de Souza, Marcela Nunes Videira, Francisco Célio Maia Chagas, Eliane Tie Oba Yoshioka, Marcos Tavares-Dias

**Affiliations:** 1 Programa de Pós-graduação em Biodiversidade Tropical – PPGBIO, Universidade Federal do Amapá - UNIFAP, Macapá, AP, Brasil; 2 Centro Universitário Metropolitano da Amazônia – UNIFAMAZ, Belém, PA, Brasil; 3 Universidade do Estado do Amapá – UEAP, Macapá, AP, Brasil; 4 Embrapa Amazônia Ocidental, Manaus, AM, Brasil; 5 Embrapa Amapá, Macapá, AP, Brasil

**Keywords:** Infection, essential oil, fish parasites, treatment, Infecção, óleo essencial, parasitos de peixe, tratamento

## Abstract

This study investigated for the first time the effectiveness of therapeutic baths with essential oil (EO) of *Piper hispidum* against monogeneans *Anacanthorus spathulatus*, *Notozothecium janauachensis*, *Mymarothecium boegeri* and *Linguadactyloides brinkmanni* from the gills of *Colossoma macropomum*, as well as the hematological and histological effects on this fish. In therapeutic baths, 100 mg/L of *P. hispidum* essential oil and two control groups (water from the culture tank and water from the culture tank with 70% alcohol) were exposed for 1 h/day, with intervals of 48 hours for 3 days, and three replicates each were used. Therapeutic baths with 100 mg/L of *P. hispidum* essential oil had an efficacy of 78.6% against monogeneans. The toxicity of this essential oil was low, since there were a few physiological and histopathological changes that did not compromise the functioning of the gills of the fish. Therefore, 100 mg/L of *P. hispidum* essential oil was effective for controlling monogeneans in *C. macropomum*, when short therapeutic baths were used without compromising the health of the exposed fish.

## Resumo

Este estudo investigou pela primeira vez, a eficácia de banhos terapêuticos com óleo essencial (OE) de *Piper hispidum* contra monogenéticos *Anacanthorus spathulatus*, *Notozothecium janauachensis, Mymarothecium boegeri* e *Linguadactyloides brinkmanni* das brânquias de *Colossoma macropomum*, bem como os efeitos hematológicos e histológicos neste peixe. Foram usados 100 mg/L de óleo essencial de *P. hispidum* e dois grupos controles (água do tanque de cultivo e água do tanque de cultivo com álcool 70%) expostos durante 1 hora por dia, com intervalos de 48 horas durante 3 dias, três repetições cada. Banhos terapêuticos com 100 mg/L de OE de *P. hispidum* tiveram uma eficácia de 78,6% contra monogenéticos. A toxicidade foi baixa, pois ocorreram poucas alterações fisiológicas e histopatológicas que não comprometeram o funcionamento das brânquias dos peixes. Portanto, houve eficácia de 100 mg/L de OE de *P. hispidum* para controle de monogenéticos em *C. macropomum*, quando usados banhos terapêuticos curtos sem prejudicar a saúde dos peixes expostos.

## Introduction

Aquaculture is the fastest-growing agricultural activity in recent decades and this global industry continues to grow (Gonzales et al., 2020; FAO, 2022). However, with the intensification of this activity, fish can become more susceptible to diseases due to stress and poor water quality, which mainly favor the proliferation of parasites that lead to diseases (Tavares-Dias, 2021). Aquaculture therefore needs technological innovations to improve the sanitary management of farmed fish, avoiding parasitic outbreaks and economic losses, which are among the main obstacles to the development of this activity in industrial scale (Luz et al., 2021; Alves et al., 2021; Malheiros et al., 2022). Thus, there is a growing search for ecologically friendly treatment, such as the use of oils derived from medicinal plants against parasites of fish (Gonzales et al., 2020; Barriga et al., 2020).

*Piper hispidum* Swartz is a medicinal plant popularly known as jaborandi or false jaborandi that is distributed in the Antilles, Central America and South America, including all Brazilian geographic regions (Orlandelli et al., 2012). Among the main chemical components of essential oil of *P. hispidum* are monoterpenes and sequiterpenes such as β-pinene, α-pinene, δ-3 carene, α-cadinol and spathulenol (Potzernheim et al., 2006; Andrade et al., 2009; Santos et al., 2018). Thus, the essential oil of this medicinal plant has been investigated as a source of new natural therapeutic products with potential antifungal, antioxidant, antiplasmodial and trypanocidal activity (Silva et al., 2014). Alves et al. (2021) demonstrated that *P. hispidum* essential oil had *in vitro* efficacy against monogeneans; however, this essential oil has not been used to control and treatment of fish parasites. In fish, the search for new anthelmintic treatments to control and treatment of parasitic diseases using bioactive principles from medicinal plants has showed beneficial results (Corral et al., 2018; Santos et al., 2018; Majolo et al., 2019; Barriga et al., 2020; Alves et al., 2021; Malheiros et al., 2022).

In Brazil, *Colossoma macropomum* Cuvier 1818 (Characiformes, Serrasalmidae) is considered the second largest scale fish in the Amazon basin, has presented problems of parasitic diseases caused by monogenean species in cultivation (Alves et al., 2019; Barriga et al., 2020; Tavares-Dias et al., 2021; Malheiros et al., 2022). These helminth parasites with a direct and short life cycle and with vertical transmission, factors that facilitate high levels of infection in intensive cultivation (Alves et al., 2019), cause lesions in the gills of *C. macropomum* that can compromise physiological processes such as breathing, excretion, acid-base balance and osmoregulation (Tavares-Dias et al., 2021). Thus, this study investigated the efficacy of therapeutic baths with essential oil of *P. hispidum* against monogeneans of *C. macropomum* gills, as well as the hematological and histological effects on this fish.

## Material and Methods

### Fish and acclimation

Fingerlings of *C. macropomum* (± 25 g) were obtained from a commercial fish farm in Macapá, state of Amapá (Brazil) and maintained at the Aquaculture and Fisheries Laboratory from Embrapa, Macapá, Amapá (Brazil). The fish were acclimatized in 500 L tanks and kept in an open water system for 10 days with constant aeration and continuous water renewal (1.1 L /min) and fed twice a day with a commercial diet containing 32% protein crude (Guabi^®^, Brazil). This fish stock naturally infected by monogeneans was used in all assays. The following water parameters were measured daily: mean temperature (29.8 ± 0.1°C), dissolved oxygen (5.5 ± 0.2 mg/L), pH (5.8 ± 0.2), ammonia (0.4 ± 0.2 mg/L), alkalinity (10.0 ± 0.001 mg/L) and hardness (11.0 ± 0.1 mg/L), using a multiparameter probe (YSI, USA). The tank was siphoned weekly to remove accumulated organic matter, and the water was renewed.

### Obtaining and chemical composition of *P.* *hispidum* essential oil

*Piper hispidum* was cultured in the Medicinal Plants and Vegetables Sector of Embrapa Western Amazon, Manaus, state of Amazonas (Brazil). The branches and inflorescence of *P. hispidum* were used to obtaining of essential oil. This essential oil was extracted through hydrodistillation with Clevenger apparatus for 4 h. The chemical composition of the essential oil was determined using gas chromatography–mass (GC-MS, Shimadzu QP5050A), following the methodology described by Adams (2007). The majority constituents of *P. hispidum* essential oil were the γ-terpineno (30.9%), α-terpineno (14.0%), *p*-cimeno (12.0%), α-selineno (9.0%), β-selineno (8.1%) e terpinoleno (7.3%) (Alves et al., 2021).

### Therapeutic baths with essential oil of *P. hispidum* in *C. macropomum*

*Colossoma macropomum* (29.08 ± 11.4 g and 11.4 ± 1.7 cm) were exposed to 100 mg/L of *P. hispidum* essential oil (Alves et al., 2021) for 1 h per day, at 48 h intervals for 3 days, using three treatments with three replicates each, with 13 fish per replicate, totaling 39 per treatment*.* Two control groups were used, one with water from the culture tank and the other with water from the culture tank + ethyl alcohol (70%). The *P. hispidum* essential oil was diluted using 70% ethyl alcohol (1:10 g) as solvent. The fish were kept in a static water system when the essential oil was added to the experimental tanks, after 1h, the water of the tanks was changed.

On the sixth day after the third therapeutic baths, the fish were euthanized by medullary section and the gills collected and fixed in 5% formalin to quantify the monogenean species (Eiras et al., 2006) and determine the prevalence and mean abundance of these parasites (Bush et al., 1997). Identification of parasites followed the recommendations of Cohen et al. (2013).

### Blood analysis in *C. macropomum* after therapeutic baths with *P. hispidum* essential oil

After the sixth day of therapeutic baths with 100 mg/L of *P. hispidum* essential oil, the blood of the fish was collected by puncturing the caudal vessel using syringes with EDTA (10%). Three treatments with three replicates each were used, with five fish per replicate, totaling 15 fish per treatment. Two control groups were used: one with water from the culture tank and other with water from the culture tank + ethyl alcohol (70%). The blood was used to determine the hematocrit, using the microhematocrit method; counts of total number of erythrocytes using a Neubauer chamber and hemoglobin concentration by the cyanmethemoglobin method. Hematimetric indices such as mean corpuscular volume (MCV) and mean corpuscular hemoglobin concentration (MCHC) were calculated from the data of erythrocytes, hematocrit and hemoglobin. Blood smears were confectioned and panchromatically stained with a May Grünwald–Giemsa–Wright combination for differential leukocyte counts in up to 200 cells of interest. Blood smears were also used to determine the total number of leukocytes and thrombocytes using indirect method (Ranzani-Paiva et al., 2013).

### Histopathological analyses of *C. macropomum* gills after therapeutic baths with *P. hispidum* essential oil

After the sixth of the third therapeutic baths with *P. hispidum* essential oil, the fish were euthanized by medullary section and the gills of nine animals per treatment (three of each replicate) were collected for histopathological analysis. The first right and left gill arches of each fish were collected and fixed in Davidson's solution (95% alcohol, formaldehyde, acetic acid and distilled water) for 48 h, then dehydrated in ascending order of alcohol (70%, 80%, 90%, absolute I, II and III), cleared in xylene concentration (100%), impregnated and embedded in paraffin, to obtain the blocks. The paraffin blocks were cut in a microtome (Leica DM 1000) with five µm thickness. After making the slides (in duplicates), they were stained with Hematoxylin and Eosin (HE). Images were taken using a common optical microscope (Leica DM 1000, USA) and the software Leica Application Suite 1.6.0 software. Histopathological analyses were performed in a semiquantitative manner using mean assessment values (MAV) (Schwaiger et al., 1997) and the histopathological alteration index (HAI) (Poleksić & Mitrović-Tutundžić, 1994).

### Statistical analyzes

The histopathological, blood and parasitic data were previously submitted to analysis to assess normality, using the Shapiro-Wilk test, using the “RVAideMemoire” package (Herve, 2023), and homoscedasticity, using the Levene test from the “car” (Fox & Weisberg, 2019). All data showed homogeneity in variance. Histopathological and blood data showed non-normal distribution, while parasite abundance data showed normal distribution. In this way, we used the Kruskal-Wallis test to evaluate the effect of the treatments on the histopathological and blood parameters of the animals, followed by the post hoc Dunn test using the “rstatix” package (Kassambara, 2023). For the parasite data, we used the ANOVA standard “aov” function of the R software, aiming to evaluate the effect of treatments on the abundance of monogeneans, and we applied Tukey's post hoc test, using the “DescTools” package (Signorell, 2023). All these analyzes were performed using the R software (R Core Team, 2022).

## Results

### Therapeutic baths and anthelmintic action of *P. hispidum* essential oil in *C. macropomum*

During therapeutic baths with 100 mg/L of *P. hispidum* essential oil, there was no fish mortality in any of the three treatments. The fish behavior were restlessness, lethargy, accelerated operculum movement and erratic swimming. In addition, there were sedation effects in fish exposed to 100 mg/L of *P. hispidum* essential oil. In all groups, during the 3 days of treatment, the fish were fed normally. All fish used in therapeutic baths had the gills naturally parasitized by monogeneans (*Anacanthorus spathulatus* Kritsky, Thatcher & Kayton, 1979; *Mymarothecium boegeri* Cohen & Kohn, 2005; *Notozothecium janauachensis* Belmont-Jégu, Domingues & Martins, 2004 and *Linguadactyloides brinkmani* Thatcher & Krytsky, 1983). In the gills of *C. macropomum*, the mean abundance of monogeneans was lower (p<0.05) in fish exposed to 100 mg/L of *P. hispidum* essential oil, when compared to controls exposed to culture tank water + 70% alcohol and culture tank water, which were similar to each other. However, the prevalence of monogeneans was from 100% in all treatments. Therapeutic baths with 100 mg/L of *P. hispidum* essential oil had efficacy of 78.6% against monogeneans of *C. macropomum* gills, but control group using culture tank water with 70% alcohol also had a low efficacy (Table 1).

**Table 1:** Efficacy and parasitological indices of monogeneans in gills of *Colossoma macropomum* exposed to *Piper hispidum* essential oil*.*

**Treatments**	**Prevalence (%)**	**Mean abundance**	**Efficacy (%)**
Water	100	31.1 ± 9.9^a^	-
Water+alcohol	100	27.1 ± 8.9^a^	12.7
100 mg/L	100	5.8 ± 2.1^b^	78.6

Different letter in the same column, indicate different by the Tukey test (p<0.05).

### Blood parameters of the *C. macropomum* exposed to *P. hispidum* essential oil

The concentrations of glucose, total protein, hematocrit, MCV, thrombocytes, lymphocytes and monocytes number were similar (p>0.05) in fish exposed to 100 mg/L of essential oil *P. hispidum*, control culture tank water and control water of the culture tank + 70% alcohol. The total number of erythrocytes, MCHC and total number of leukocytes decreased (p<0.05) in control groups using culture tank water and culture tank water + 70% alcohol, but treatment with *P. hispidum* essential oil were similar (p>0.05) to the two control groups. Hemoglobin and number of basophils were similar (p>0.05) between the control groups with water from the culture tank and water from the culture tank + 70% alcohol, but in the treatment with 100 mg/L of *P. hispidum* essential oil were lower (p<0.05) than in the control group with water from the culture tank. The number of neutrophils in the control groups was similar (p>0.05) and in the group exposed to 100 mg/L of *P. hispidum* essential oil was higher (p<0.05) than in the control group with culture tank water + 70% alcohol. The number of eosinophils and PAS-GL in the controls with culture tank water + 70% alcohol and treatment with 100 mg/L of *P. hispidum* essential oil were similar (p>0.05), however they were lower (p<0.05) that in the control using water from the culture tank (Table 2).

**Table 2:** Blood parameters of *Colossoma macropomum* exposed to *Piper hispidum* essential oil.

**Parameters**	**Water**	**Water + alcohol**	**100 mg/L**
Glucose (mg/dL)	81.3 ± 20.7^a^	77.8 ± 13.6^a^	92.9 ± 43.1^a^
Total protein (g/dL)	2.9 ± 0.5^a^	2.5 ± 0.4^a^	2.6 ± 0.4^a^
Erythrocytes (x10^6^/μL)	1.57 ± 0.27^a^	1.23 ± 0.29^b^	1.35 ± 0.31^a,b^
Hematocrit (%)	25.57 ± 4.15^a^	26.17 ± 2.51^a^	24.0 ± 4.3^a^
Hemoglobin (g/dL)	7.87 ± 0.67^a^	7.11 ± 0.63^a,b^	6.7 ± 1.4^b^
MVC (fL)	169.33 ± 43.45^a^	162.33 ± 41.37^a^	169.7 ± 40.9^a^
MCHC (g/dL)	31.45 ± 4.66^a^	27.26 ± 2.21^b^	27.8 ± 2.2^a,b^
Thrombocytes (μL)	170.4 ± 32.4^a^	152.3 ± 45.6^a^	137.4 ± 32.0^a^
Leukocytes (μL)	157,714 ± 27,906^a^	122,417 ± 29,039^b^	135,530 ± 31.511^a,b^
Lymphocytes (μL)	49,940 ± 21,417^a^	47,440 ± 19,317^a^	59,766 ± 26,776^a^
Monocytes (μL)	77,529 ± 28,705^a^	77,529 ± 28,705^a^	79,149 ± 30,911^a^
Neutrophils (μL)	38,490 ± 7704^a,b^	32,235 ± 8771^a^	44,594 ± 12,438^b^
Eosinophils (μL)	4537 ± 2213^a^	1632 ± 1243^b^	834 ± 1132^b^
PAS-GL (μL)	32,564 ± 11,290^a^	14,335 ± 5545^b^	15,244 ± 7635^b^
Basophils (μL)	2360 ± 2080^a^	928 ± 983^a,b^	821 ± 931^b^

Data express mean ± deviation standard. Different letter, in the same line, indicate difference by the Dunn test (p<0.05). PAS-GL: Positive-PAS granular leukocytes; MCHC: Mean corpuscular hemoglobin concentration; MCV, Mean corpuscular volume.

### Histopathological analyses of *C. macropomum* gills after therapeutic baths with *P. hispidum* essential oil

**Figure 1:**
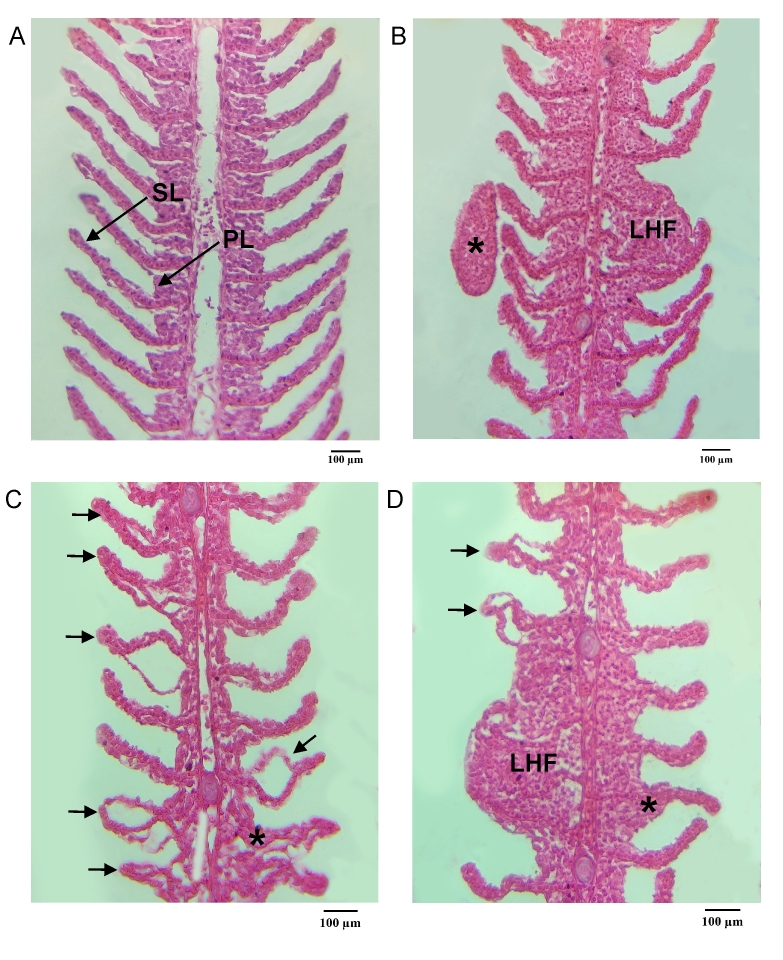
Histopathology of the gills of *Colossoma macropomum*. A-B. Gills of fish exposed to 100 mg/L of *Piper hispidum* essential oil*.* A. Fish gills showing primary (PL) and secondary (SL) lamellae (arrow). B. Gill filaments showing lamellar hyperplasia with fusion (LHF) and monogenean (asterisk). C. Gills of fish exposed to culture tank water + 70% alcohol (control) showing detachment of the lamellar epithelium (arrow) and lamellar hyperplasia (asterisk). D. Gills of fish exposed from culture water (control) showing detachment of the lamellar epithelium (arrow), lamellar hyperplasia (asterisk) and lamellar hyperplasia with fusion (LHF).

In the fish of group exposed to 100 mg/L of *P. hispidum* essential oil, hyperplasia with lamellar fusion and the presence of monogenean were observed (Figure 1A-B). In the control group using tank water + 70% alcohol there was detachment of the lamellar epithelium and lamellar hyperplasia (Figure 1C). In the control group with water from the culture tank, there were discreet histopathological changes in the gills, such as detachment of the lamellar epithelium, lamellar hyperplasia and lamellar hyperplasia with fusion (Figure 1D).

In fish exposed to 100 mg/L of *P. hispidum* essential oil, culture tank water + 70% alcohol and culture tank water, the mean MAV values were similar (p>0.05). However, the HAI values in the control group with tank water + 70% alcohol were higher (p<0.05) than in fish exposed to culture tank water and exposed to 100 mg/L of essential oil of *P. hispidum* (Table 3).

**Table 3:** Mean assessment values (MAV) and values of histopathological alteration index (HAI) for gills of *Colossoma macropomum* submitted to therapeutic baths with *Piper hispidum* essential oil*.*

Treatments	N	MAV	HAI	Severity of the lesions according to the HAI
Water	9	4.0 ± 2.4^a^	4.5 ± 4.2^a^	Normal functioning of the organ
Water + alcohol	9	6.0 ± 2.0^a^	11.8 ± 4.1^b^	Mild to moderate of the organ damage
100 mg/L	9	5.2 ± 2.2^a^	5.3 ± 4.2^a^	Normal functioning of the organ

Data express mean ± deviation standard. Different letters in the same line indicate differences significant between treatments (p<0.05) by the by the Dunn test.

## Discussion

In recent years, with the growing demand for fish as food, aquaculture needs innovative therapeutic interventions to overcome the challenges in terms of developing adequate technologies for management of diseases in cultivation systems (Malheiros et al., 2022). Essential oils are mixtures of volatile metabolites that usually consist of several different bioactive compounds and in varying concentrations. Hence, the bioactive effects of essential oils are often due to the synergy of their bioactive compounds (Tavares-Dias, 2018; Barriga et al., 2020; Alves et al., 2021), many of which consist of compounds that induce anesthesia in the fish (Barriga et al., 2020; Aydın & Barbas, 2020; Alves et al., 2021), as it happened with essential oil of *P. hispidum* (Alves et al., 2021). However, an adequate anesthetic should quickly immobilize the fish, result in an uneventful, irreversible recovery, and should be high potency and widely available, cost-effective, and have low or no toxicity.

Currently, the use of essential oils has been a growing alternative in the control and treatment of infections caused by monogeneans in fish (Soares et al., 2016; Tavares-Dias, 2018; Barriga et al., 2020). Alves et al. (2021) reported *in vitro* efficacy (100%) of 250, 350, 600 and 800 mg/L of *P. hispidum* essential oil against monogeneans of *C. macropomum*. Therapeutic baths with 100 mg/L of essential oil of *P. hispidum* had a high efficacy against monogeneans of *C. macropomum* gills. In addition, the treatment using culture tank water with 70% alcohol (control) had a low efficacy, which has also been reported in other previous studies (Tavares-Dias, 2018; Barriga et al., 2020).

In fish, as in other vertebrate animals, studies of blood constituents can be used as prognostic indicators of pathological conditions, especially when considering changes in sick animals, in which the severity of changes implies poor conditions during challenges imposed as for example, stress and toxicity (Soares et al., 2016; Ranzani-Paiva et al., 2013). In *C. macropomum*, hemoglobin, number of eosinophils, PAS-GL and basophils were decreased in fish exposed to 100 mg/L of essential oil of *P. hispidum*. However, there were no changes in MCV and MCHC and, therefore, this anemia can be characterized as normocytic-normochromic. Anemia due to reduced hemoglobin level also occurred after exposure of *C. macropomum* to therapeutic baths with 700 mg/L of *Lippia grata*, due to stress (Barriga et al., 2020). It has been shown that baths with 100 mg/L of oleoresin from *Copaifera reticulata* caused a reduction in the number of eosinophils in *C. macropomum* (Malheiros et al., 2022). Eosinophils participate in defense processes against infection caused by parasites (Ranzani-Paiva et al., 2013). According to Martins et al. (2009), PAS-LG migrates to the inflammatory focus, although its participation in this process is still unclear.

In *C. macropomum*, exposed to culture tank water with 70% alcohol, there was a reduction in MCHC, total number of erythrocytes and leukocytes and number of neutrophils, which may be related to a possible effect of toxicity on fish. Erythrocytes are cells whose function is to transport respiratory gases by binding to hemoglobin (Ranzani-Paiva et al., 2013). In *C. macropomum*, exposed to culture tank water with 70% alcohol was also reported decrease in MCHC (Barriga et al., 2020). This reduction in the total number of leukocytes and neutrophils may indicate a decrease in immunity, as these defense cells, in general, respond to parasitic infection and stress in fish (Urbinati et al., 2020; Yonar et al., 2020). In addition, this reduction in the number of leukocytes due to the decrease in the number of neutrophils may also be related to damages to the branchial lamellar epithelium due to exposure to 70% alcohol. A reduction in the total number of leukocytes was also reported in *C. macropomum* submitted to therapeutic baths with 300 mg/L of *Alpinia zerumbet* essential oil (Luz et al., 2021) and with 250 mg/L of *C. reticulata* oleoresin nanoemulsion (Malheiros et al., 2022), whose branchial epithelium was also affected.

In the gills of *C. macropomum* exposed to 100 mg/L of *P. hispidum* essential oil, hyperplasia with lamellar fusion was observed. In the gills of fish exposed to culture tank water + 70% alcohol (control), there was detachment of the lamellar epithelium and lamellar hyperplasia, while in fish kept with water from culture tank (control) there was detachment of the lamellar epithelium, lamellar hyperplasia and lamellar hyperplasia with fusion, probably due to also the presence of monogeneans (Tavares-Dias et al., 2021). However, in *C. macropomum*, these lesions varied from mild to moderate, resulting in little impairment of gill functions, but that did not affected the normal functioning of the gills, according to the degree of severity of the lesions (Schwaiger et al., 1997). On the gills of *C. macropomum*, baths with 100 mg/L of *C. reticulata* oleoresin or with 250 mg/L of *C. reticulata* nanoemulsion caused epithelial detachment, lamellar hyperplasia and hypertrophy, resulting in moderate fusion of secondary lamellae (Malheiros et al., 2022). Similar structural alterations were also reported in the gills of *C. macropomum* caused both by the toxicity of the essential oil *Lippia alba* and the diluent used, the 70% ethyl alcohol (Soares et al., 2016), as well as in the gills of this fish exposed to 300 mg/L of *A. zerumbet* essential oil (Luz et al., 2021).

In conclusion, three short therapeutic baths with 100 mg/L of *P. hispidum* essential oil, with intervals of 48 h, showed a good efficacy (78.6%) against monogeneans, indicating the application of this concentration to control of these parasites in *C. macropomum*. Furthermore, therapeutic baths with *P. hispidum* essential oil caused few physiological and histopathological changes in fish*.*
