# ERRATUM

**DOI:** 10.1590/S1984-29612025008

**Published:** 2025-02-03

In the article “Essential oil of *Piper hispidum* (Piperaceae) has efficacy against monogeneans, and effects on hematology and gill histology of *Colossoma macropomum*” (DOI https://doi.org/10.1590/S1984-29612024001) published in issue 1, volume 33, 2024, the Brazilian Journal of Veterinary Parasitology, e014723, which reads:

Francisco Célio Maia Chagas

Read up:

Francisco Célio Maia Chaves

